# Validation of the Reference Genes for the Gene Expression Studies in Different Cell Lines of Pig

**DOI:** 10.1155/2021/5364190

**Published:** 2021-08-18

**Authors:** Chun-Di Xie, Bingyuan Wang, Zhao-Ji Shen, Wen-Ye Yao, Hong Ao, Bugao Li, Yangli Pei, Rong Zhou

**Affiliations:** ^1^State Key Laboratory of Animal Nutrition; Key Laboratory of Animal Genetics Breeding and Reproduction, Ministry of Agriculture and Rural Affairs, Institute of Animal Science, Chinese Academy of Agricultural Sciences, 100193 Beijing, China; ^2^Guangdong Provincial Key Laboratory of Animal Molecular Design and Precise Breeding, College of Life Science and Engineering, Foshan University, 528231 Foshan, China; ^3^College of Animal Science, Shanxi Agricultural University, 030801 Shanxi, China

## Abstract

Reverse transcription quantitative real-time polymerase chain reaction is one of the important methods to investigate gene expression in cells and tissues. However, if the data cannot be normalized with appropriate reference genes, the results may be unreliable. In this study, we detected the expression of 15 reference genes in three pig cell lines. The results showed that *SDHA* and *ALDOA* were the most stable reference genes in 3D4/21 cells. *TOP2B*, *TBP*, and *PPIA* were the most stable reference genes in PK-15 cells. *SDHA* and *ALDOA* were the most stable reference genes in IPEC-J2 cells. In addition, each cell line only needs to use two reference genes to standardize the expression of target genes. Taken together, this study provides a reference for different pig cell lines to select reference genes and also provides a theoretical basis for the use of these cell lines in related functional researches.

## 1. Introduction

Reverse transcription quantitative real-time polymerase chain reaction (RT-qPCR) is frequently used to detect gene expression in cells and tissues due to its high sensitivity, specificity, and accuracy [1–4]. However, the results of RT-qPCR can be affected by some factors, including varying quality and quantity of RNA, different sample amounts, enzymatic efficiency in reverse transcription steps, and PCR amplification efficiency [5, 6]. Therefore, it is necessary to select a proper reference gene as an internal control to correct and normalize the expression of target gene. Excellent reference genes need to be expressed stably under all conditions. Glyceraldehyde 3-phos-phate dehydrogenase (GAPDH), *β*-actin (ACTB), and 18S ribosomal RNA (18S) are considered to be stable expression in different conditions and tissues and are widely used as reference genes [7]. However, it has been proved that the expression of these genes was not as stable as initially thought. Many studies showed that these genes had a great different expression level in different experimental conditions [8–15]. Therefore, it is necessary to select suitable reference genes according to different tissues, cells, and experiments.

Pig cell lines are often used as cell models to explore the mechanism of gene function and immune disease. Porcine alveolar macrophages (3D4/21 cells), isolated from the lung of Landrace pig and immortalized with SV40 large T antigen transformed with pSV3neo, could secrete cytokines and were used to study the mechanism of Streptococcus suis [16] and swine fever virus [17]. Porcine kidney 15 cell line (PK-15) is a clone of PK-1a cell, which is often used to study circovirus [18]. And intestinal porcine epithelial cells (IPEC-J2) are most commonly used to study porcine epidemic diarrhea-related diseases [19]. In these researches, a large number of gene expression verification are usually involved, and the inaccurate results of RT-qPCR will lead to the failure of the experiment. Therefore, it is of great importance to select an appropriate reference gene for these cell lines. At present, most of the studies on reference genes are focused on tissues, but few on cell lines, especially on these three types of pig cell lines.

The aim of this study was the selection of suitable reference genes for expression studies in pig cell lines using quantitative RT-qPCR. In this study, the expression level of 15 candidate genes in porcine 3D4/21, PK-15, and IPEC-J2 cell lines was detected. The expression stability of genes was analyzed and evaluated by geNorm [6], NormFinder [20], and BestKeeper [21]. The findings screened out the most stable reference genes for these three porcine cell lines and provided the reference for carrying out relevant experiments to select reference genes.

## 2. Materials and Methods

### 2.1. Cell Culture

PK15, IPEC-J2, and 3D4/21 cells were seeded at the concentration of 1 × 10^5^ cells per well in 12 well plates containing 1 mL of DMEM (Gibco, 12800082) or RPMI-1640 (Gibco, 42401042), respectively, and supplemented with 10% fetal bovine serum (ExCell Bio, FND500). Cells were cultured in a highly humidified atmosphere of 95% air and 5% CO_2_ at 37°C.

### 2.2. Isolation of RNA and Real-Time Quantitative PCR (RT-qPCR)

Cell were homogenized in 500 *μ*L of RNA isolater (Vazyme, R401-01) according to the manufacturer's instructions, purified by DNaseI, and quantified by spectrophotometry. Each cell had three biological repetitions. The cDNA for qPCR analysis was synthesized using the HiScript III 1st Strand cDNA Synthesis Kit (+gDNA wiper) (Vazyme, R312-01). Prior to qPCR amplification, cDNA was diluted to 300 ng/*μ*L. The reaction mixture for the qPCR step was ChamQ Universal SYBR qPCR Master Mix (Vazyme, Q711-02). The 15 *μ*L RT-qPCR reaction mixture encompassed 1 *μ*L of cDNA template, 7.2 *μ*L of 2 × SYBR premix Ex Taq, 0.3 *μ*L of 50 × ROX Reference Dye II, 0.3 *μ*L of each forward and reverse primer, and 5.9 *μ*L of double-distilled water. Each sample was performed in triplicates. And the RT-qPCR conditions were as follows: 95°C for 5 min, followed by 40 cycles of 95°C for 5 s and 60°C for 34 s. RT-qPCR was performed using TB Green Premix Ex Taq in a QuantStudio 3 (Thermo Fisher Scientific, QuantStudio 3).

### 2.3. Reference Gene Selection

The candidate reference gene with stable level of expression and similar transcript number in the cells were chosen [1, 22]. The 15 pairs of primer sequences from literature or NCBI used in this study are listed in Table 1.

### 2.4. Data Analysis

The expression level of all genes was converted into relative expression level 2 ^-*Δ*CT^ (ΔCT = CT sample − CT minimum). Then, these data were imported into geNorm (v3.5) and NormFinder.

The geNorm program calculates the average stable expression value (*M*, value) of each reference gene to select the gene with better stability. The genes with high *M* values are less stably expressed and would not be proper reference genes. On the contrary, the genes with low *M* values are stably expressed and would be suitable reference genes. The software can also calculate the paired variations (*V*) of the standardized factor by increasing a new reference gene and determine the optimal number according to the VN/VN + 1 value. The gene-stability measure in geNorm for control gene is the arithmetic mean of all pairwise variations [6].

NormFinder can calculate the stable value of reference gene expression, and the criterion is same with geNorm [20]. However, the program can only select the most suitable genes.

The BestKeeper (version 1) program can directly calculate the CT value of gene expression. The program can obtain the correlation coefficient (*R*), standard deviation (SD), and coefficient of variation (CV) of pairing between each gene. In this program, reference genes with high *R* value and low CV and SD value are more stable [21].

## 3. Results

### 3.1. The Expression of Candidate Genes in Three Cell Lines Detected by RT-qPCR

**Figure 1:**
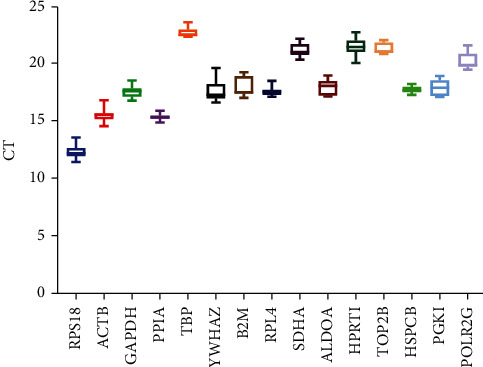
Box-and-whisk plot displaying the range of CT values of three cell lines for each reference gene. Note: different colors represent different genes. The median was marked by the line in the box.

The expression of 15 candidate genes was detected by RT-qPCR. The CT value was used to detect the stability of gene expression (Figure 1). The results showed that the average CT value of *RPS18* was the lowest (12.33), while the average CT value of TATA box binding protein (*TBP*) was the highest (22.65). These candidate genes are highly expressed in all cell lines, but their expression level varies greatly. Therefore, it is necessary to evaluate the stability of gene expression and determine the appropriate number of internal reference genes for accurate gene expression profile analysis in different cells.

### 3.2. Expression Stability of Candidate Genes Analyzed by geNorm

**Figure 2:**
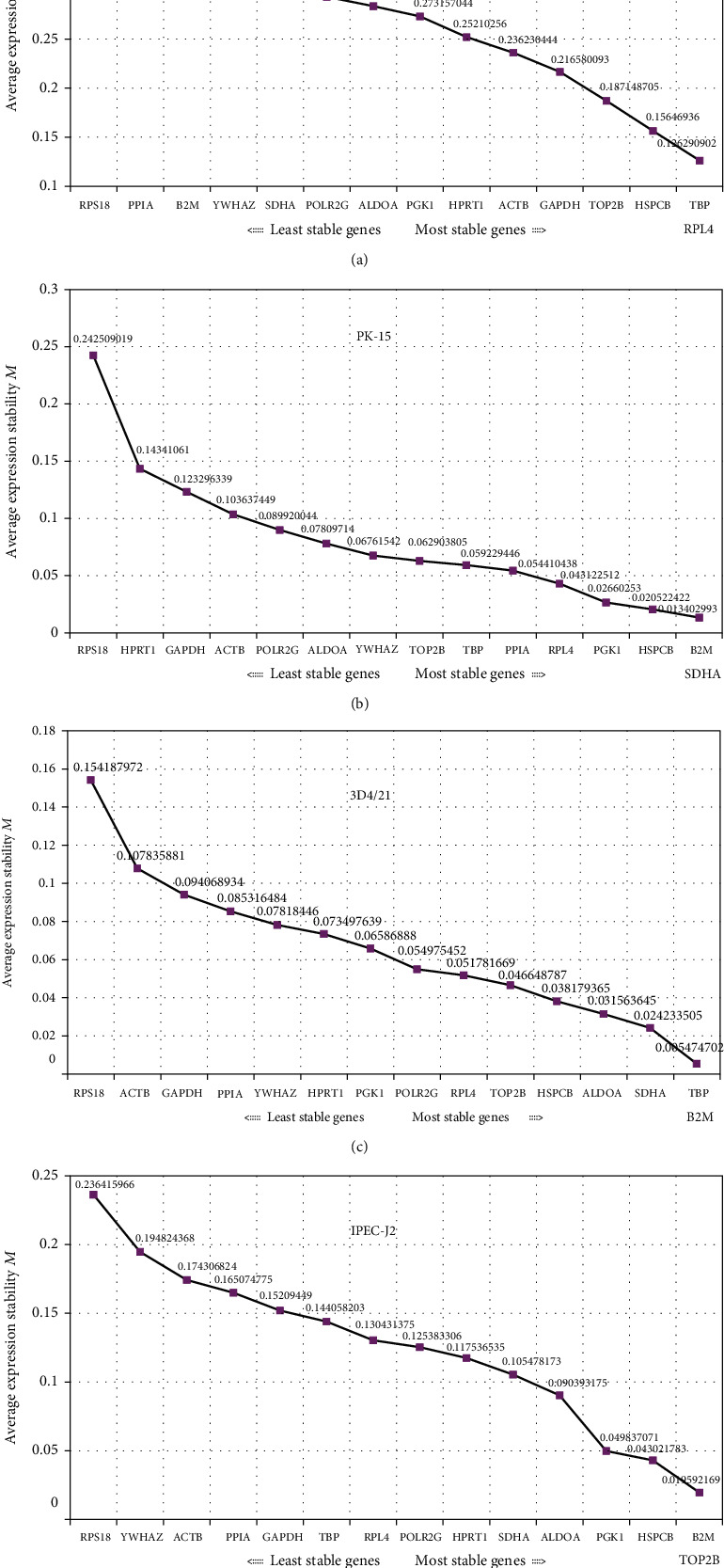
Average expression stability (*M*) of reference genes by geNorm: (a) average expression stability (*M*) of all cell types; (b) average expression stability (*M*) of PK-15; (c) average expression stability (*M*) of 3D4/21; (d) average expression stability (*M*) of IPEC-J2.

The *M* values of 15 candidate genes were calculated by geNorm (v3.5) program. The results are shown in Figure 2. The order of gene stability in all cell lines is (from the most stable to the least stable): Ribosomal protein L4(*RPL4*)/*TBP*, Heat shock 90 kDa protein 1, beta (*HSPCB*), Topoisomerase II beta(*TOP2B*), *GAPDH*, *ACTB*, Hypoxanthine phosphoribosyl transferase 1 (*HPRT1*), Phosphoglycerate kinase 1 (*PGK1*), Aldolase A, fructose-bisphosphate (*ALDOA*), Polymerase (RNA) II (DNA directed) polypeptide G (*POLR2G*), Succinate dehydrogenase complex, subunit A (*SDHA*), Tyrosine 3-monooxygenase/tryptophan 5-monooxygenase activation protein, zeta polypeptide (*YWHAZ*), beta-2-microglobulin (*B2M*), Peptidylprolyl isomerase A (cyclophilin A) (*PPIA*), and *RPS18* (Figure 2(a)). In PK-15 cell line, *SDHA* and *B2M* were the most stable genes, and *RPS18* was the least stable gene (Figure 2(b)). *B2M* and *TBP* with low *M* values were identified as the two most stable genes in 3D4/21 cell line (Figure 2(c)). *B2M* and *TOP2B* which had low *M* values were identified as the two most stable genes in IPEC-J2 cell line (Figure 2(d)). Specially, *RPS18* was the least stable in any cell types.

**Figure 3:**
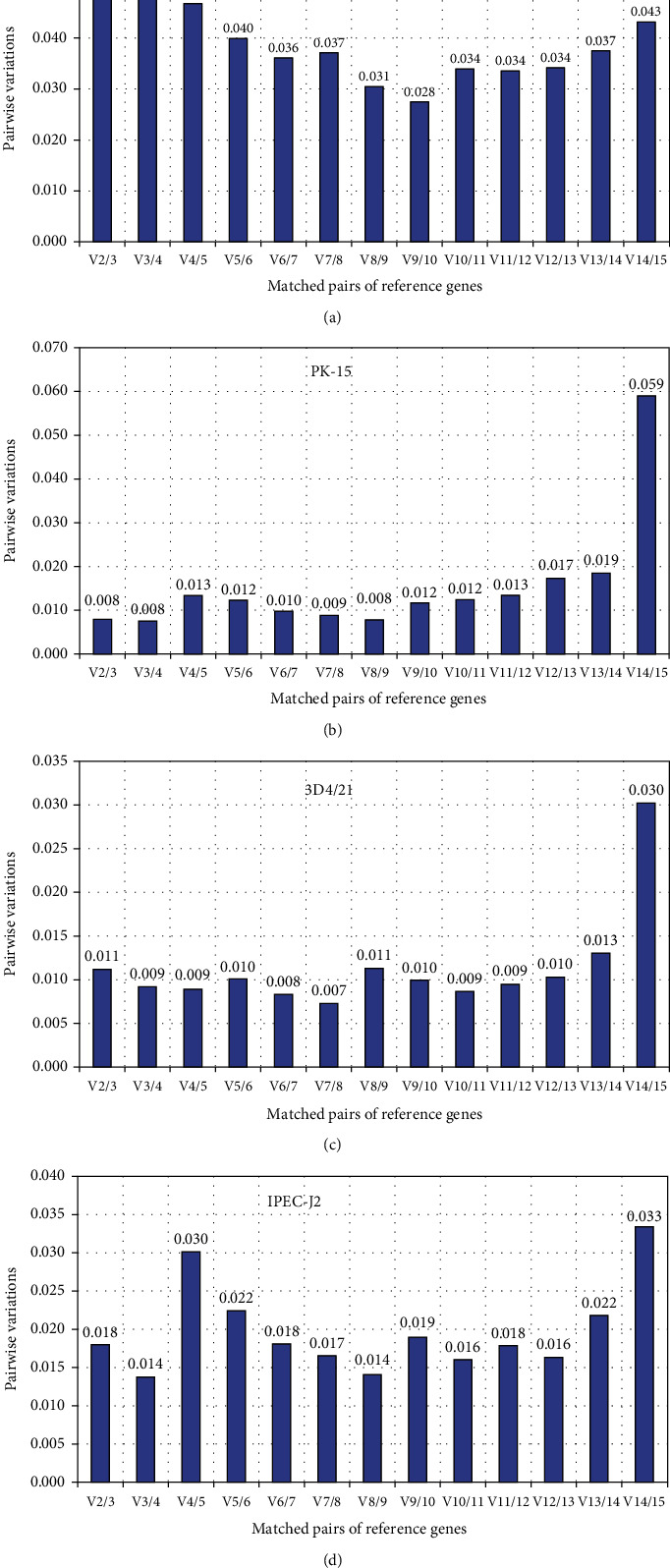
Determination of the optimal number of reference genes: (a) the optimal number of reference genes for all cells; (b) the optimal number of reference genes for PK-15; (c) the optimal number of reference genes for 3D4/21; (d) the optimal number of reference genes for IPEC-J2.

One reference gene is usually not enough for gene expression analysis in all cell types. Therefore, geNorm is used to analyze the optimal number of reference genes. GeNorm computationally introduces a new reference gene. Then, the paired variation *V* value of the factor was standardized. Thereafter, the optimal number of reference genes was determined by the value of VN/VN + 1. If the value of VN/VN + 1 is less than 0.15, *N* is the most suitable reference gene number. The results showed that VN/VN + 1 was less than 0.15 in all three cells, so the two reference genes were the best combination for gene expression analysis of the three cells (Figure 3(a)). The optimal number of reference genes for PK-15, 3D4/21, and IPEC-J2 cells is shown in Figures 3(b)–3(d),and the number is two.

### 3.3. Analysis of Gene Stability Value by NormFinder Program

The NormFinder program calculated the stability of gene expression (stability value (SV)), which was used to rank the genes. According to NormFinder analyses (Table 2), we found that *POLR2G* was the most stable in 3D4/21 cells. *PPIA* and *TBP* were the most stable in PK-15 cells. *POLR2G* and *RPL4* were the most stable in IPEC-J2 cells. These results were not completely consistent with the results analyzed by geNorm. Maybe the algorithms of the two software are different. Although the two algorithms are different, *RPS18* was the least stable in both software.

### 3.4. Analysis of Gene Stability Value by BestKeeper Program

The BestKeeper program is an Excel-based software tool. On the basis of the correlation coefficients (*R*), CV, and SD values, the optimal reference gene was determined (Table 3). The optimal reference gene was selected by high *R* value (≥0.900) and low CV and SD value. This program can calculate *R* values for up to 10 genes. Therefore, we selected 10 reference genes for analysis according to the results of geNorm and NormFinder. In all samples with higher *R* values (≥0.900), the *SDHA* and *ALDOA* genes had the lowest CV and SD values in 3D4/21 cells, the *TOP2B* gene had the lowest CV and SD values in PK-15 cells, and the *PGK1* gene had the lowest CV and SD values in IPEC-J2 cells. These results indicate that the most stable reference genes in 3D4/21, PK-15, and IPEC-J2 are *SDHA/ALDOA*, *TOP2B*, and *PGK1*, respectively.

## 4. Discussion

RT-qPCR is one of the most commonly used methods in molecular biology research for gene expression. Appropriate reference genes are very important for the reliability and repeatability of gene expression results. However, the idealized reference gene does not exist. Therefore, it is necessary to select suitable reference genes under different experimental conditions. At present, the selection of reference genes has been studied in different species, experimental conditions, cells, and tissues. However, no systematic analysis of suitable endogenous control genes exists for different types of pig cell lines.

At present, there are only some studies in human cells. RefFinder was used to select the optimal reference genes of human reticulocyte [28]; MPP1 and GAPDH were predicted as the best reference genes of reticulocyte through comprehensive sequencing. The reference genes of nine hepatocellular carcinoma (HCC) cell lines were systematically evaluated, revealing that TFG and SFRS4 were the most reliable reference genes [29].

As an increasing study exploring mRNA expression in pig cells has been published, there has been a greater interest in evaluating the commonly used, widely expressed housekeeping genes for comparisons between different types of pig cells. The expression stability of four genes (*EEF1A1*, *GAPDH*, *HPRT1*, and *TOP2B*) in pig tissues was evaluated by geNorm [30], and the results showed that *EEF1A1* and *TOP2B* were the most stable genes in kidney tissues, while *HPRT1* and *TOP2B* were the most stable genes in lung tissues. However, in our study, the result showed that *B2M* and *SDHA* were the most stable reference genes in PK-15 cells, which isolated from kidney tissues. *TBP* and *B2M* were the most stable reference genes in 3D4/21 cells, which is different from the results in corresponding tissues, indicating that the reference genes suitable for tissues may not be the right housekeeping genes in cells. Furthermore, even though *SDHA* and *ALDOA* in 3D4/21 cells, *TOP2B*, *TBP*, and *PPIA* in PK-15 cells, and *SDHA* and *ALDOA* in IPEC-J2 cells are recommended as reference gene in our study, the PK-15 cells and IPEC-J2 cells, which are both epithelial cells, owned dissimilar recommended reference genes.

In addition, the ranking of reference genes was different according to the geNorm NormFinder and BestKeeper analyses. *B2M/TBP*, *SDHA/B2M*, and *TOP2B/B2M* were the most stable reference genes in 3D4/21, PK-15, and IPEC-J2, by geNorm, respectively. NormFinder identified that the expression of *POLR2G* in 3D4/21, *PPIA/TBP* in PK-15, and *POLR2G/RPL4* in IPEC-J2 was highly stable. BestKeeper evaluates the expression variation for each single reference gene and showed that the most stable reference genes were *SDHA/ALDOA* in 3D4/21, *TOP2B* in PK-15, and *PGK1* in IPEC-J2. The published work conducted on reference genes in 7 different porcine tissues has analyzed the stability of 15 candidate genes (*ACTB*, *GAPDH*, *HPAR1*, *ALDOA*, *B2M*, *HSPCB*, *PPIA*, *PGK1*, *POLR2G*, *RPL4*, *TBP*, *RPS18*, *SDHA*, *TOP2B*) by NormFinder, geNorm, and BestKeeper, and the results were also inconsistent, which may lead by the software algorithm deviation [1]. As we all know, instead of *SDHA*, *ALDOA*, *TOP2B*, *TBP*, and *PPIA*, *ACTB*, *GAPDH*, and *RPS18* were the most popular reference genes in the mRNA expression-related study; it may due to the lack of systematic analysis of suitable endogenous control genes that exists under different conditions.

## 5. Conclusion

This investigation found evidence that there can be variation in the expression of commonly used reference genes in different type of pig cells. In general, we recommend taking *SDHA* and *ALDOA* in 3D4/21 cells, *TOP2B*, *TBP*, and *PPIA* in PK-15 cells, and *SDHA* and *ALDOA* in IPEC-J2 cells as reference genes to normalize the mRNA expression level.

## Figures and Tables

**Table 1:** Primers for the candidate reference genes and their parameters derived from RT-qPCR data analysis.

Gene	Gene name	Primer sequence (5′-3′)	Amplicon size (bp)	Reference
*B2M*	Beta-2-microglobulin	F: TTCACACCGCTCCAGTAG R: CCAGATACATAGCAGTTCAGG	166	[23]
*TBP*	TATA box binding protein	F: GATGGACGTTCGGTTTAGG R: AGCAGCACAGTACGAGCAA	124	[23]
*YWHAZ*	Tyrosine 3-monooxygenase/tryptophan 5-monooxygenase activation protein, zeta polypeptide	F: ATGCAACCAACACATCCTATC R: GCATTATTAGCGTGCTGTCTT	178	[24]
*PPIA*	Peptidylprolyl isomerase A (cyclophilin A)	F: CACAAACGGTTCCCAGTTTT R: TGTCCACAGTCAGCAATGGT	171	[25]
*RPL4*	Ribosomal protein L4	F: TTGGCATCGCAGAGTGAA R: CAGAACAGCCTCCTTGGT	178	NCBI
*SDHA*	Succinate dehydrogenase complex, subunit A	F: CTACAAGGGGCAGGTTCTGA R: AAGACAACGAGGTCCAGGAG	141	[22]
*HPRT1*	Hypoxanthine phosphoribosyl transferase 1	F: CCGAGGATTTGGAAAAGGT R: CTATTTCTGTTCAGTGCTTTGATGT	181	[23]
*TOP2B*	Topoisomerase II beta	F: AAGGGCGAGAGGTCAATGAT R: ACATCTTCTCGTTCTTGCGC	115	[1]
*ALDOA*	Aldolase A, fructose-bisphosphate	F: GAACCAACGGCGAGACAA R: ATGATGGCGAGGGAGGAG	142	[26]
*HSPCB (HSP90AB1)*	Heat shock 90 kDa protein 1, beta	F: GGCAGAAGACAAGGAGAAC R: CAGACTGGGAGGTATGGTAG	131	[26]
*PGK1*	Phosphoglycerate kinase 1	F: AGATAACGAACAACCAGAGG R: TGTCAGGCATAGGGATACC	126	[27]
*POLR2G*	Polymerase (RNA) II (DNA directed) polypeptide G	F: CTCAAGTCAACAAGGTCGGAC R: GTCCCAACAATCTTCAGGCG	181	[1]
*ACTB*	Beta actin	F: GGACTTCGAGCAGGAGATGG R: AGGAAGGAGGGCTGGAAGAG	138	NCBI
*GAPDH*	Glyceraldehyde-3-phosphate dehydrogenase	F: TCGGAGTGAACGGATTTGGC R: TGACAAGCTTCCCGTTCTCC	189	NCBI
*RPS18*	Ribosomal protein S18	F: GTAACCCGTTGAACCCCATT R: CCATCCAATCGGTAGTAGCG	151 bp	NCBI

**Table 2:** Analysis of gene stability value by NormFinder program.

All three cells		3D4/21		PK-15		IPEC-J2	
Gene	Stability value	Gene	Stability value	Gene	Stability value	Gene	Stability value
*RPL4*	0.04	*POLR2G*	0.006	*PPIA*	0.005	*RPL4*	0.020
*TOP2B*	0.07	*RPL4*	0.008	*TBP*	0.005	*POLR2G*	0.020
*TBP*	0.07	*SDHA*	0.009	*TOP2B*	0.007	*GAPDH*	0.066
*HSPCB*	0.08	*ALDOA*	0.009	*RPL4*	0.008	*ALDOA*	0.066
*POLR2G*	0.1	*HSPCB*	0.010	*YWHAZ*	0.022	*SDHA*	0.076
*GAPDH*	0.11	*TOP2B*	0.026	*PGK1*	0.026	*PPIA*	0.086
*HPRT1*	0.11	*TBP*	0.030	*HSPCB*	0.041	*HPRT1*	0.088
*ACTB*	0.12	*B2M*	0.034	*POLR2G*	0.052	*TBP*	0.091
*YWHAZ*	0.12	*PPIA*	0.061	*ALDOA*	0.057	*ACTB*	0.103
*ALDOA*	0.13	*PGK1*	0.080	*B2M*	0.057	*HSPCB*	0.125
*PGK1*	0.14	*HPRT1*	0.088	*SDHA*	0.063	*PGK1*	0.126
*SDHA*	0.16	*YWHAZ*	0.092	*ACTB*	0.080	*TOP2B*	0.152
*B2M*	0.18	*ACTB*	0.105	*GAPDH*	0.178	*B2M*	0.164
*PPIA*	0.19	*GAPDH*	0.110	*HPRT1*	0.218	*YWHAZ*	0.183
*RPS18*	0.22	*RPS18*	0.314	*RPS18*	0.613	*RPS18*	0.346

**Table 3:** Analysis of gene stability value by BestKeeper program.

3D4/21	*PPIA*	*TBP*	*B2M*	*RPL4*	*SDHA*	*ALDOA*	*TOP2B*	*HSPCB*	*PGK1*	*POLR2G*
*n*	3	3	3	3	3	3	3	3	3	3
Geo mean	15.33	22.43	17.47	17.27	20.95	17.18	20.91	17.47	17.15	19.64
Std dev	0.10	0.02	0.01	0.07	0.04	0.03	0.05	0.06	0.03	0.08
CV	0.64	0.08	0.08	0.42	0.18	0.20	0.26	0.32	0.19	0.42
*r*	0.873	0.816	0.408	1.030	0.913	0.913	0.862	0.862	-0.913	0.936

PK-15	*PPIA*	*TBP*	*YWHAZ*	*B2M*	*RPL4*	*SDHA*	*TOP2B*	*HSPCB*	*PGK1*	*ALDOA*
*n*	3	3	3	3	3	3	3	3	3	3
Geo mean	15.38	22.44	17.21	17.22	17.45	20.54	21.01	17.73	17.90	18.09
Std dev	0.07	0.08	0.09	0.14	0.10	0.14	0.06	0.13	0.11	0.08
CV	0.45	0.34	0.55	0.79	0.56	0.67	0.28	0.71	0.62	0.43
*r*	0.998	0.950	0.858	0.971	0.973	0.985	0.966	0.968	0.995	0.581

IPEC-J2	*ACTB*	*GAPDH*	*B2M*	*RPL4*	*SDHA*	*ALDOA*	*TOP2B*	*HSPCB*	*PGK1*	*POLR2G*
*n*	3	3	3	3	3	3	3	3	3	3
Geo mean	15.93	17.99	18.96	17.91	21.82	18.59	21.84	17.99	18.63	21.04
Std dev	0.36	0.30	0.09	0.26	0.20	0.20	0.11	0.13	0.12	0.24
CV	2.29	1.65	0.48	1.44	0.90	1.05	0.49	0.69	0.66	1.16
*r*	1.000	0.988	0.853	0.992	0.965	0.982	0.869	0.991	0.997	0.998

## Data Availability

The data used to support the findings of this study are available from the corresponding author upon request.
